# 

**Published:** 1867-12

**Authors:** 


### CONTENTS.

### ORIGINAL COMMUNICATIONS.

PAGE.
ARTICLE I.—Is Belladona an Antidote to Opium? By W. C. BELLAMY, Columbus, Georgia ........ 415
ARTICLE II.—Proceedings of Atlanta Medical College........ 419
ARTICLE III.—Singular Species of Epizoa. By N. B DREWRY, M. D., Griffin, Ga... 425

### SELECTIONS.

Cases by J. W. THOMPSON, M. D., of Paducah, Ky........ 426
Letter to Dr. E. S. FRAZER, from his son, S. H. FRAZER, in Paris.—The General Pathology of Syphilis—Letter III.—The Indurated Chancre........ 431

### EDITORIAL AND MISCELLANEOUS.

Bibliographical........ 446

European Correspondence........ 456

To our Readrrs........ 460

